# Notes of Hospital Practice

**Published:** 1872-11

**Authors:** Frank Woodbury

**Affiliations:** Reporter


### SELECTIONS.

---

### NOTES OF HOSPITAL PRACTICE—JEFFERSON MEDICAL COLLEGE.

*CLINIC OF PROF. S. D. GROSS.—Reported by Frank Woodbury.*

*Hœmatoid Tumor*—September 28.—This young German, twenty-two years of age, was brought here by his physician from Mauch Chunk, Pennsylvania, on account of a tumor on the anterior aspect of the right elbow-joint. This, he tells us, has existed for eighteen months, and he first noticed it about three months after receiving a severe blow with a stick, upon the part, which bruised and broke the skin. This tumor has been persistent, gradually attaining the size of a hen's egg, and deprived him of the use of his arm by the pain which was produced by moving it. Eight weeks ago his physician commenced an operation for the removal of the tumor. He made a subcutaneous exploratory incision, by entering a bistoury under the skin about an inch farther down the arm and running it up into the swelling. This was followed by profuse hemorrhage, which, although finally controlled by cold applications, deterred him from proceeding further. This sinus has remained open, and the flow of blood recurring at short intervals has rendered the patient almost exsanguineous; his face is anæmic and pallid.

We find here œdema of the hand and forearm, undoubtedly produced by this circumscribed tumor in the bend of the elbow, which, by pressing on the superficial veins, obstructs the return of the blood from the parts and forces some of its watery constituents through the coats of the smaller vessels. On examining this tumor we find it circumscribed, with considerable hardness and some fluctuation, and a sensation as if it contained a semi-fluid substance. This inclines me to the opinion that it may be of a nævoid character or a blood-cyst, caused by the injury. It is not an aneurism of the brachial artery, as it is not in the line of that vessel, and there is no pulsation or aneurismal thrill. A grooved director passes readily up the sinus into the swelling, and its withdrawal is followed by the flow of a few drops of a ropy, glutinous, grumous fluid.

What I propose to do, after administering chloroform, is to insert a grooved direcror into the sinus and slit up the skin as far as the tumor. I am not prepared to state positively the exact nature of this affection, whether it is aneurismal or nævoid, malignant or benign; my object is to prevent future hemorrhage, which, if allowed to continue unchecked, would undoubtedly prove fatal. The indication is, therefore, to cut down and remove the cause if possible. We will tie any vessels we meet, as we proceed, as he is not in the best condition for the operation and can ill afford to lose any more blood. We find the parts in the neighborhood very vascular, and all the small vessels are enlarged; ligatures are of no use, as the blood flows, not from one or two, but literally oozes from a thousand points. During the operation the brachial artery is compressed by an assistant. The tendon of the biceps muscle passes directly over the tumor, and must, therefore, be divided in order to enucleate the cyst. The sac of the tumor, which is quite thick and contains a number of enlarged veins, is evidently formed by interstitial deposit in the connective tissue, which sends fibrous bands into the interior, resembling the musculi pectinati in the ventricles of the heart. Its contents are partly fluid blood, and the remainder is grumous and clotted, proving its traumatic organ.

The wound, which is quite deep, we will now fill with cotton wadding dipped in Monsel's solution, which has powerful styptic and antiseptic qualities. A roller bandage will be applied upon the hand and forearm, and the limb kept elevated. He shall have an anodyne immediately; we will give him half a grain of morphia, which is much better in these cases than smaller doses. In order to keep down inflammation and avert hemorrhage, ice will be applied to the part, for the same reason that ice is applied to the head in cerebral meningitis, to reduce the heat and morbid action; the same care, however, must be exercised not to allow it to remain too long in contact with the surface, lest it should freeze the part and produce sloughing. He must remain in bed for a day or two, until the danger from hemorrhage is over; to avoid this, as the brachial artery may he extensively diseased, tournaquet will be loosely applied around the arm, ready to be used in an emergency. As his system is in such a depressed condition, we will bring it up with a nourishing diet, with animal broths and a glass of milk-punch every day, and the administration of iron and quinine.

Twenty-five years ago, when bleeding was much more frequently performed than it is now, effusions of blood in the cellular tissue, with the formation of a bloody cyst, or hæmatoma, as it is now called, quite commonly resulted from venesection in the arm. Occasionally the lancet pierces the artery and forms an aneurism. In such a case the best plan of treatment would be to cut down upon the artery and tie it above and below the lesion. I remember, when in Louisville, a man came to me whose arm, in consequence of a venesection, had become swollen and dark-colored from the wrist to the axilla. Erysipelas had ensued, exhausting the patient and threatening his life. I ligated the artery above and below the point of injury, laid the parts open freely, turned out the blood, and saved the man. The young practitioner who attended him was afterwards mulcted in heavy damages, although I do not think that he was to blame, as such an accident might follow any venesection.

October 19.—The wound has entirely filled up with granulations. The styptic cotton was allowed to remain in the wound for three days, at which time suppuration commenced and the healing process began. He has been using the oxide of zinc ointment, which is especially adapted to cases where there is irritation of the skin, such as itching or pruritus, but does not possess any cicatrizing qualities. We will therefore suspend its use, and apply instead the acid nitrate of mercury ointment, which is a powerful cicatrizer ; this should, however, never be used in the full strength as sold in the shops, which is too stimulating, but should be diluted in the proportion of one part of the ointment to ten parts of lard, and be applied on a piece of lint.

The muscles are stiff from want of use and from interstitial deposits. The inflammation resulting from the operation has caused an effusion of plasma among the muscles, and has extended into the joint, producing false anchylosis. To overcome this, passive motion must be used twice in the twenty-four hours, performed gently at first, so as not to interfere with the healing process or produce fresh inflammation. The limb is to be washed daily with warm water and a little soap, so as to stimulate absorption. The patient has much improved since the operation, both in appearance and condition. The treatment, with daily open-air exercise, will be continued for a short time until he is perfectly well.

*An Unusual Case of Hydrocele.*—This interesting specimen was furnished by Dr. Barton, chief of my surgical clinic. The patient from whom it was obtained, a man thirty-five years of age, has been troubled for several succeeding summers by a great increase in the size of his scrotum, which, during the winter months, gradually resumed its natural condition. This summer it was more distended than ever. In June, Dr. Barton drew off with the trocar eight fluid-ounces of a fluid resembling milk in color and consistency; three weeks later seven ounces were removed, and on the 12th of this month six ounces more, somewhat darker in color; which is here exhibited.

The fluid of hydrocele, especially in young subjects, is as a rule, a pale, watery liquid, but sometimes of a pale sherry color. This always contains a large amount of albumen, which coagulates by heat. In rare cases, by reason of its containing in addition a quantity of fatty matters, especially of a micaceous-looking substance known as cholesterin, it assumes this color. From a careful analysis of the fluid in this case, made by Dr. Barton, I am enabled to give you its chemical composition. It was found to contain sulphate of soda, tri-basic phosphate of soda, chloride of sodium, urea, albumen, fat, and water. On microscopical examination the field was filled with fat-globules, which were entirely soluble in ether.

*Spermatorrhœa.*—This patient, a young man of twenty-two years, has suffered from frequent seminal losses during the past eight or nine years; the primary cause he considers was masturbation. The discharges occur several times a week during sleep, and he also thinks that some passes off in the urine. He is troubled with indigestion and constipation, is depressed in spirits, and his general health is undermined.

This is a not unfrequent result from a practice which, I regret to say, is not exclusively confined to the male sex, and is generally acquired while at school. The continued irritation of the genital organs induces an erethism of the entire genito-urinary tract, the parts being in a state of unnatural excitation. This determines more blood to the organs, and slight causes are sufficient to induce erections and seminal losses, generally at night. At first these emissions occur during sleep, towards morning, accompanied by a lascivious dream and sensation. Ultimately, as the parts become enfeebled, the discharge occurs without sensation, consciousness, or erection, not being discovered until the succeeding morning, or it may even occur during straining at defecation. This may continue for years, ruining the health and depressing the mind, especially if the exciting cause is continued. The individual constantly broods over his condition ; he cannot look his fellow-man in the face, and shuns society ; if he sees a friend coming towards him in the street, he will, in all probability, cross to the other side to avoid him. This may end in settled melancholy, epilepsy, or insanity.

This affection can only be confounded with prostatorrhœa, which consists in the discharge of a few drops of mucus from the prostate gland and urethra, taking place either in expelling the final drops of urine, during defecation, or from excitement. Emissions taking place in the daytime are generally of this character, while those at night are true seminal fluid. But seminal losses, within physiological limits, do not constitute a disease. Every man in health has occasional emissions, which generally occur during sleep, under the influence of a dream. It is only when these become abnormally frequent that the disorder is called spermatorrhœa, affects the patient's health, and calls for treatment. His statement that he lost the fluid during urination is incorrect. It never passes in that way, as is proven by a microscopical examination of the urine; but if there was an actual mingling of the two fluids, this element would be present in much larger quantity.

Where the case is plainly one of spermatorrhœa, treatment should be instituted promptly. Milder cases will generally recover spontaneously after withdrawal of the exciting cause, by proper attention to the diet, avoiding anything of a stimulant character, and invigorating the system by cold baths and exercise. In chronic cases the best treatment is cauterization of the prostatic sinuses, to remove the hyperæsthesia, of the ejaculatory ducts. These are in a state of morbid sensibility, and our object is to remove this condition so as to lead them back to their natural condition. An instrument was devised for this purpose by Lallemand of Montpellier, who wrote a book on this subject. His instrument consists of a catheter, from whose extremity a cup, containing a stick of lunar caustic, is made to project by pushing in a stylet at the handle. One objection to this instrument is that in several cases, in which it was used, the caustic broke off in the urethra and gave trouble. To obviate this, I modified the instrument, many years ago, by having it closed at the vesical extremity, near which, on its convex surface, is a fenestra, which is placed over the desired spot, and a little cup containing nitrate of silver is brought opposite it and in contact with the part, by pushing in the stylet. This instrument we will now use, introducing it as an ordinary catheter until we have reached the proper situation, where it is allowed to remain for a few moments, and is then withdrawn slowly, sa as to extend the impression along the urethra. After this operation there will be some pain and scalding, and anodyne should be given. I generally prefer in these cases two drachms of paregoric or one-sixth of a grain of morphia. The operation may be repeated at the end of eight or nine days; it must not be performed too frequently, as it would do harm. Three or four applications, in most cases, are all that will be required to effect a cure. All sources of irritation should be avoided. The bowels should be kept in a soluble condition by some aperient laxative, and the general health improved by tonics, open-air exercise, cold bathing, and a nourishing diet.

*Syphilitic Ulceration of the Face.*—This young married woman has a number of superficial ulcers involving the upper lip and entire nose, which is partly destroyed. These extend from the mouth, are covered with spoiled lymph, and tell their own story. The patient lives with her husband, who is healthy, and she denies ever having the primary lesion, sore throat, or eruption on the skin, but I am, nevertheless, convinced that it is nothing but syphilitis. Why it selected the face in preference to the legs or the skin instead of the lymphatic glands, hair, periosteum, or bones, no one can tell. You can judge positively from its appearance that this is not cancerous, as it has not the aspect of an epitheliomatous sore.

Now, what should be done here in the way of treatment?

To treat it locally, only, would be the height of folly; we will, therefore, also give our constitutional remedies. The iodides, either of potassium, sodium, or ammonium, produce almost a specific effect in this disease, especially when combined, as I am in the habit of using them, with a small quantity of the bichloride of mercury. Nausea, produced either by too large doses or a too long continuance of them, must be avoided, as it would depress the patient. We commence, then, with a small dose, and will increase it judiciously.

℞.—Potassii iodid., gr. viij;

Hydrarg. chlorid. corros., gr. $\frac{1}{10}$. M.

Take either directly before, or an hour after, each meal. The diet must be light and nutritious, and the skin must be kept clean, avoiding taking cold. As a local application to promote cicatrization,—

℞.—Liq. hydrarg. nitrat., gr. x;
Aquæ, f ℥ j. M.

Use once a day, and have a cloth wet with—

℞.—Liq. plumbi subacetat., f ℥ j,

Infus. ulmi, f ℥ viij, kept constantly on the part.

September 28.—Patient presented; much improved. Treatment continued.

October 19.—The ulcers have entirely healed under the treatment, thus verifying our diagnosis. The specific remedies will, however, be continued for some time longer, until the poison is effectually controlled; or the ulceration may return. The patient's nose is much disfigured, and at a future period, when she has quite recovered, we will make her a new nose by a plastic operation, taking a flap of skin from the adjacent parts. This is an ancient operation, and was invented and practised by the Chinese centuries ago. Tagliacozzi, an eminent Italian surgeon, by an ingenious but tedious process, took a flap from the inner side of the patient's arm, over the flexor muscle, the hand being fixed to the top of the head and the arm firmly held in position by apparatus, until the skin adhered to the edges of the gap, which had been freshened previous to stitching on the new integument. This was subsequently modified by the German surgeons, but the original Chinese method is the one now generally preferred.—*Philadelphia Medical Times.*

